# Supplementary figures and images for: Principles of RNA recruitment to viral ribonucleoprotein condensates in a segmented dsRNA virus
Source: eLife. 2023 Jan 26;12:e68670. doi: 10.7554/eLife.68670 (PMC9925054; doi:10.7554/eLife.68670)

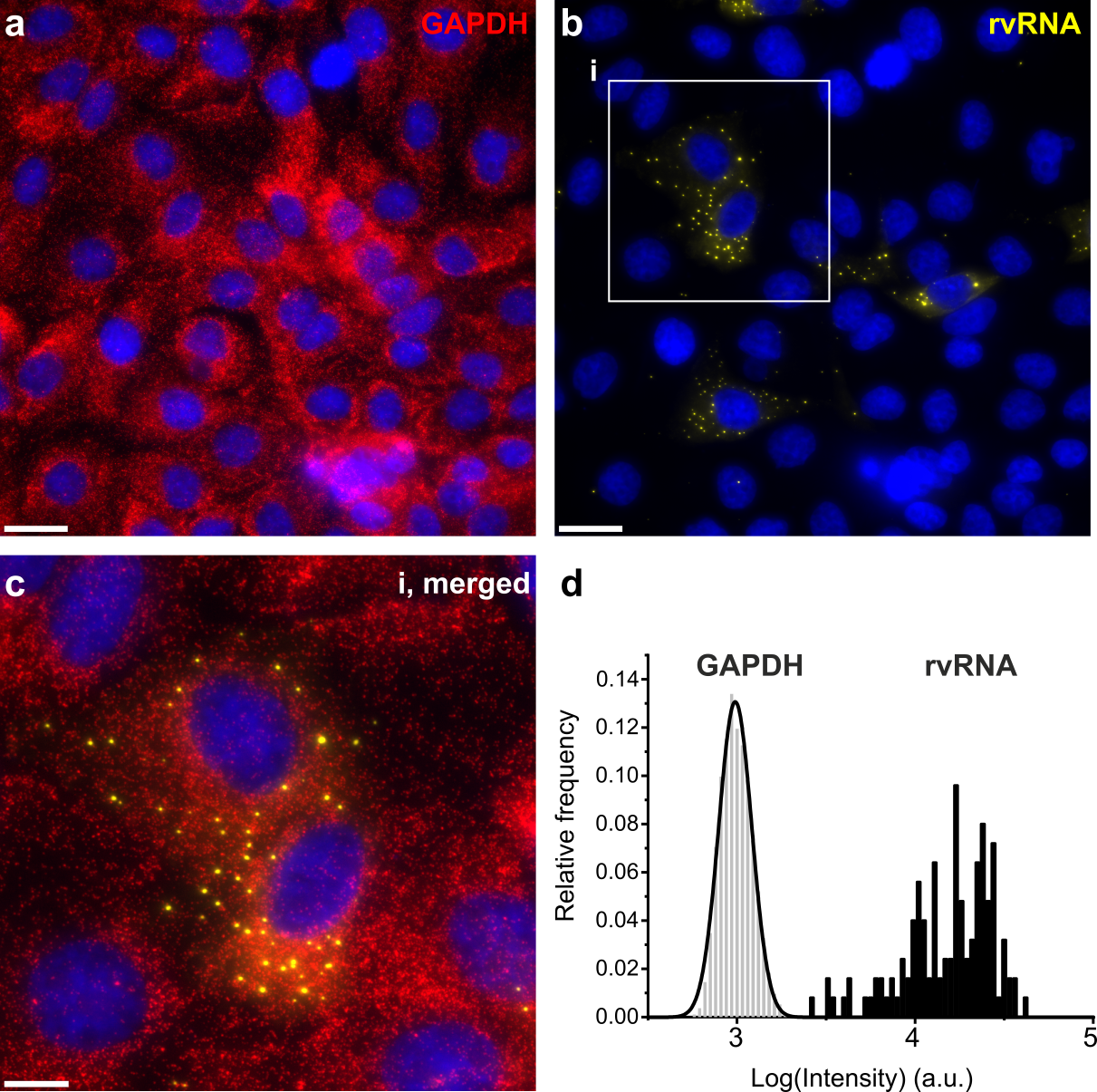

Supplement: Figure 1—source data 2. [file elife-68670-fig1-data2.zip › Fig1 - source_data2_amended_19Jan2023/Fig1 - suplementary figure 1.tif]

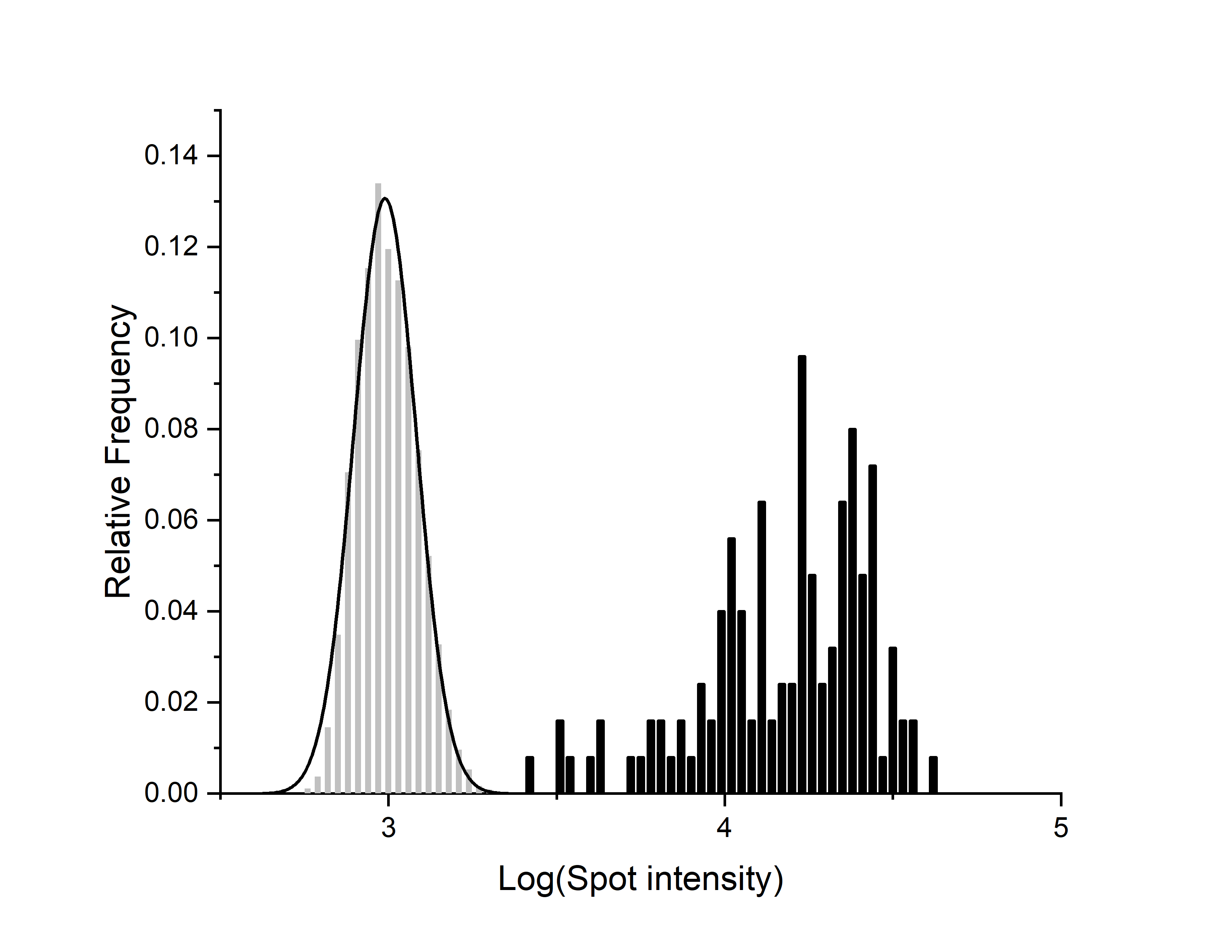

Supplement: Figure 1—source data 2. [file elife-68670-fig1-data2.zip › Fig1 - source_data2_amended_19Jan2023/Graph2.png]

- Seg4
- EGFP

## Data2 -Fig 2 - source data 6 - 6HPI

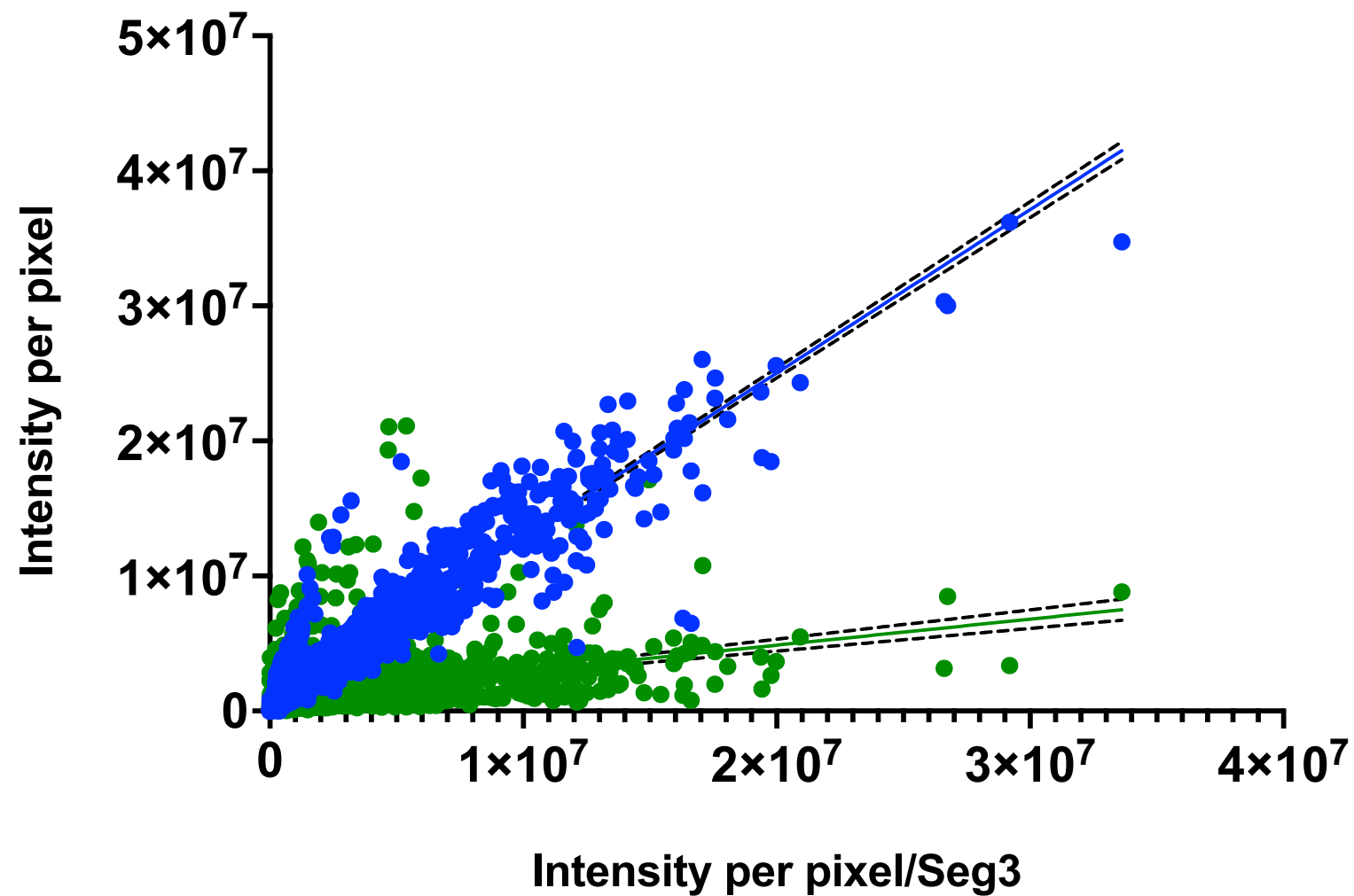

Supplement: Figure 2—source data 2. [file elife-68670-fig2-data2.zip › Fig2 - source_data2_amended_19Jan2023/Fig 2 - source data 6.pdf]

● Seg4  
■ EGFP

## Data 1 - Fig.2 - source data 2

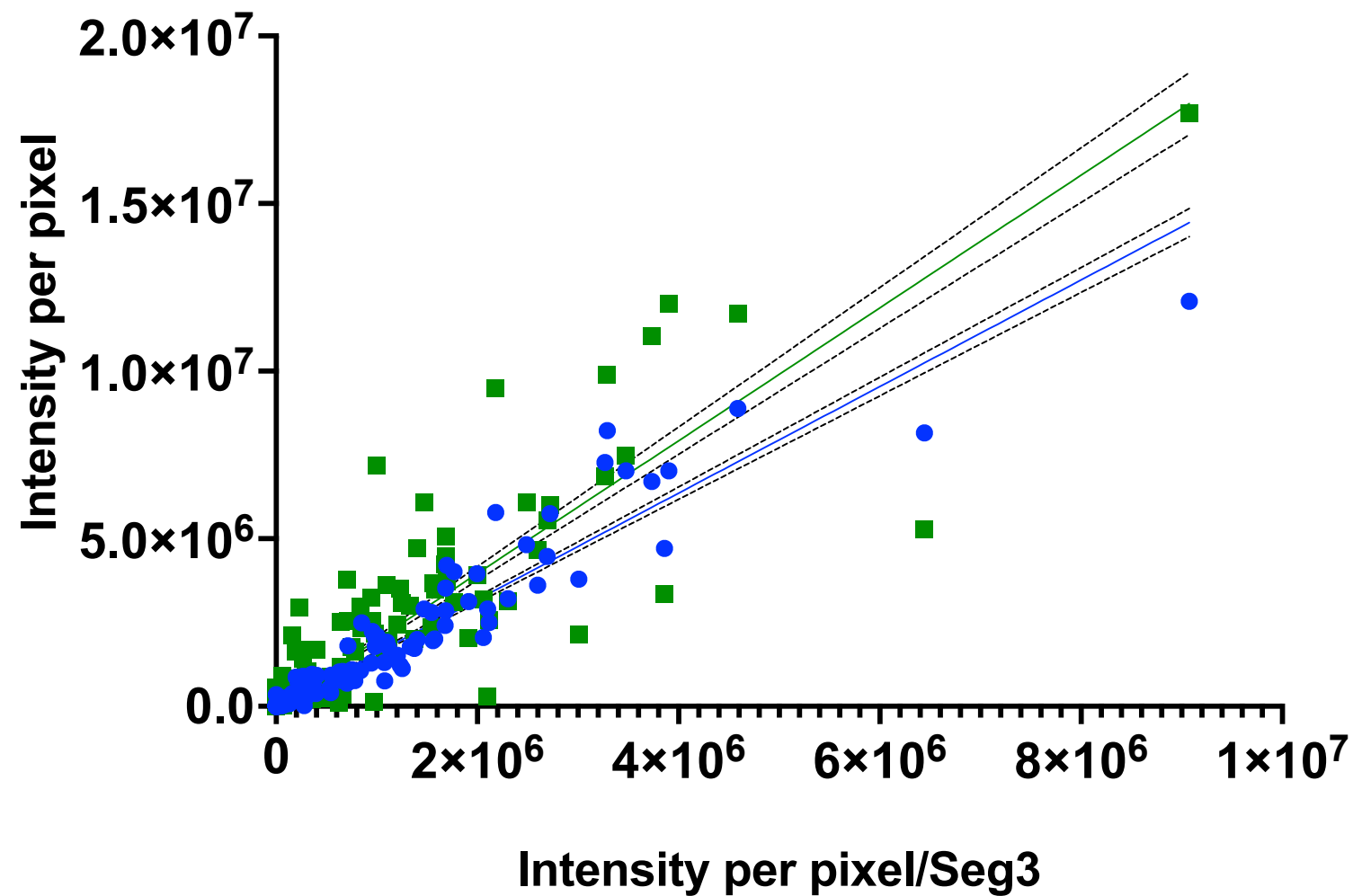

Supplement: Figure 2—source data 2. [file elife-68670-fig2-data2.zip › Fig2 - source_data2_amended_19Jan2023/ Fig2 - source data 2.pdf]

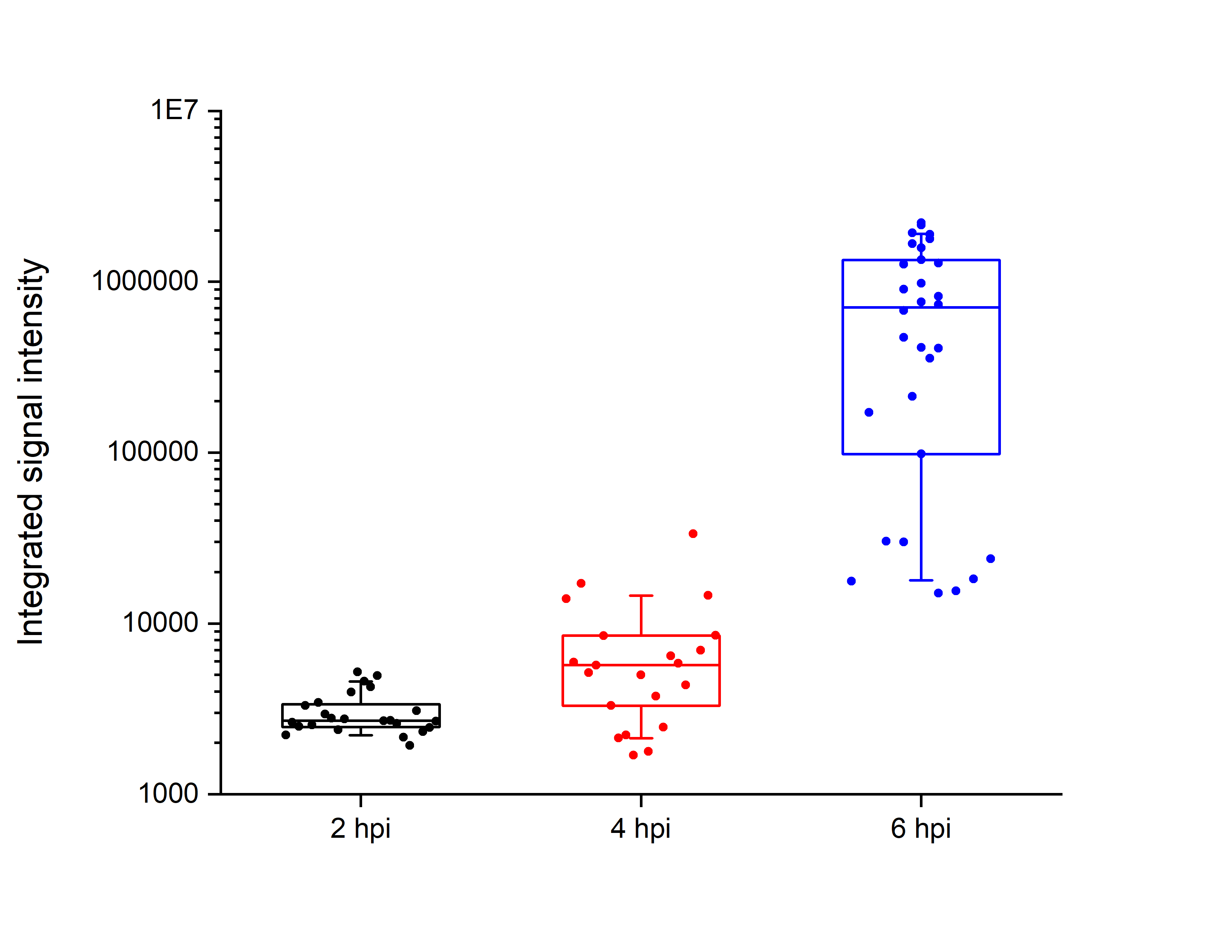

Supplement: Figure 2—source data 4. — Integrated signals were calculated for single cells separately, N=24 (2 hpi), N=21 (4 hpi), N=30 (6 hpi). [file elife-68670-fig2-data4.zip › Fig2 - source_data4_amended_19Jan2023/Seg3 RNA.png]

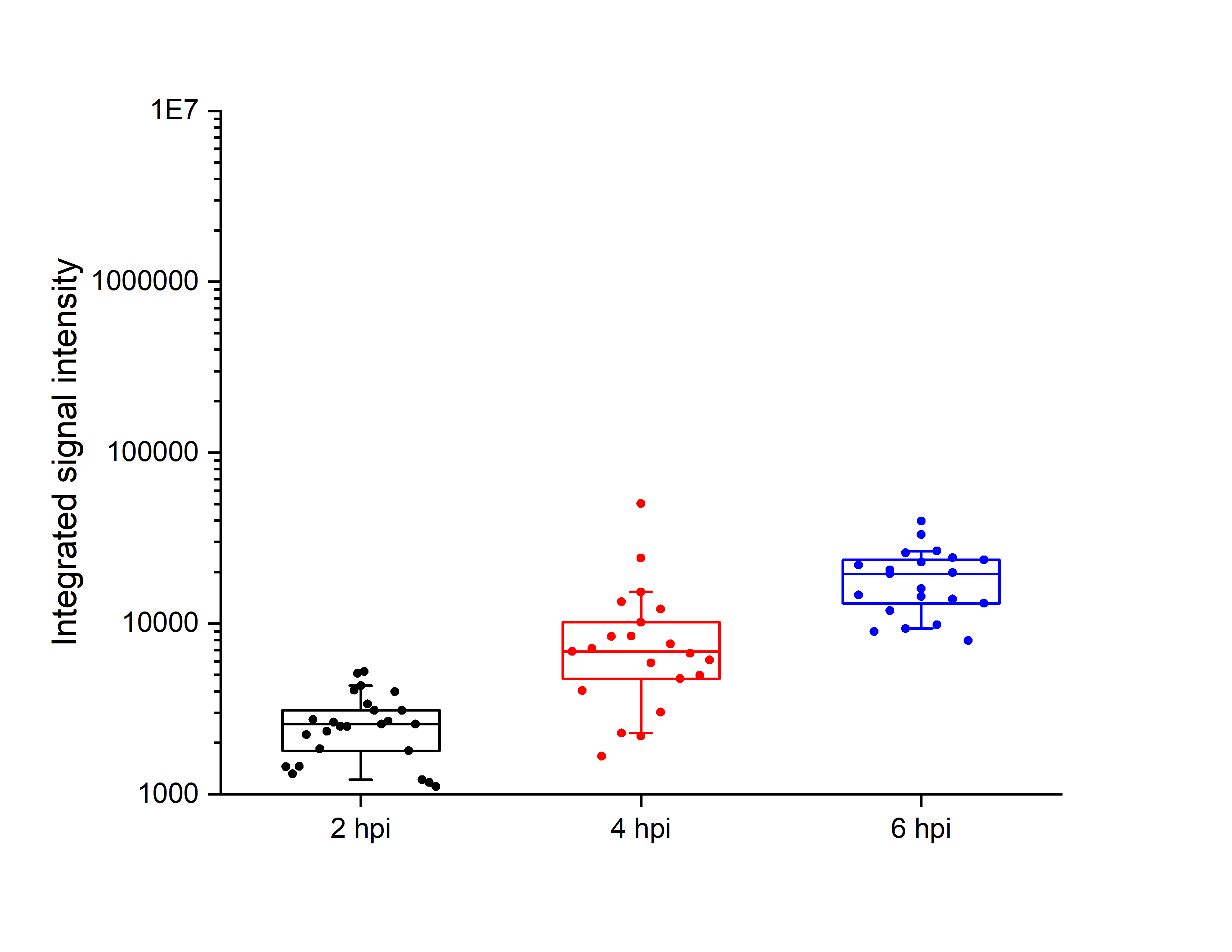

Supplement: Figure 2—source data 4. — Integrated signals were calculated for single cells separately, N=24 (2 hpi), N=21 (4 hpi), N=30 (6 hpi). [file elife-68670-fig2-data4.zip › Fig2 - source_data4_amended_19Jan2023/EGFP.png]

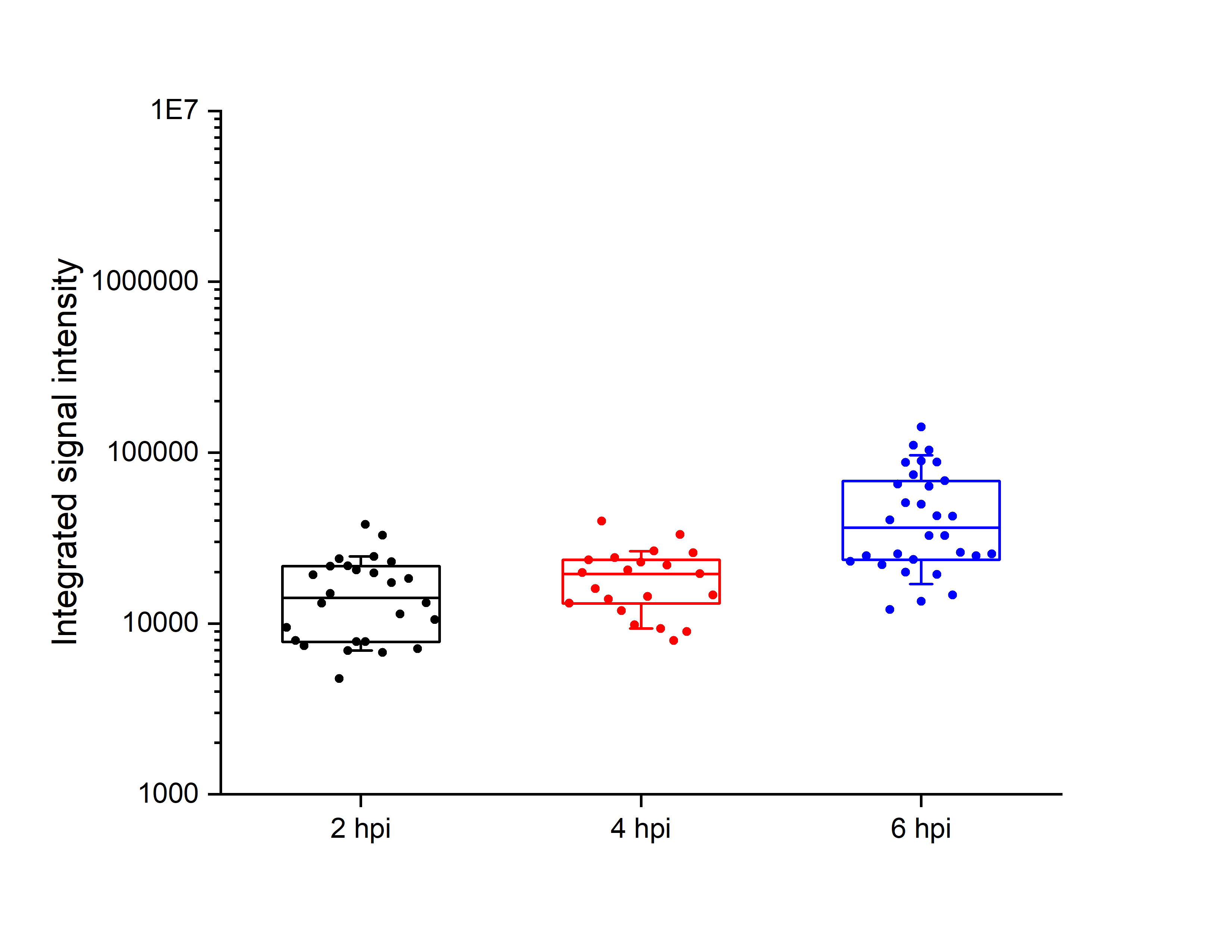

Supplement: Figure 2—source data 4. — Integrated signals were calculated for single cells separately, N=24 (2 hpi), N=21 (4 hpi), N=30 (6 hpi). [file elife-68670-fig2-data4.zip › Fig2 - source_data4_amended_19Jan2023/Seg4 RNA.png]

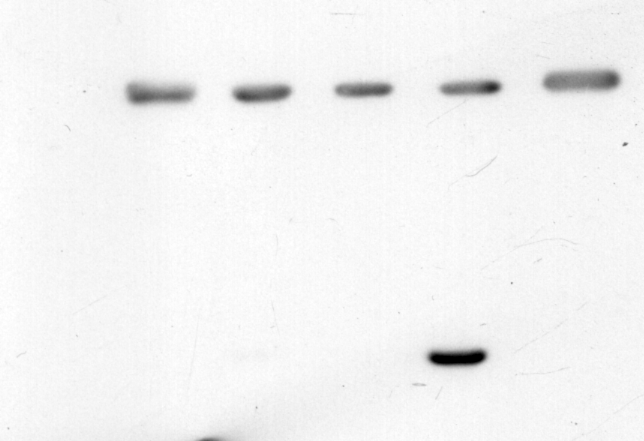

Supplement: Figure 2—source data 8. — MA104 cells stably expressing shRNA targetting NSP2 transcripts were infected at MOI = 1, and cell lysates were analysed for NSP2 expression by western blotting at 8 hpi. [file elife-68670-fig2-data8.zip › Fig2 - source data 8.tif]

# UDEX-FISH

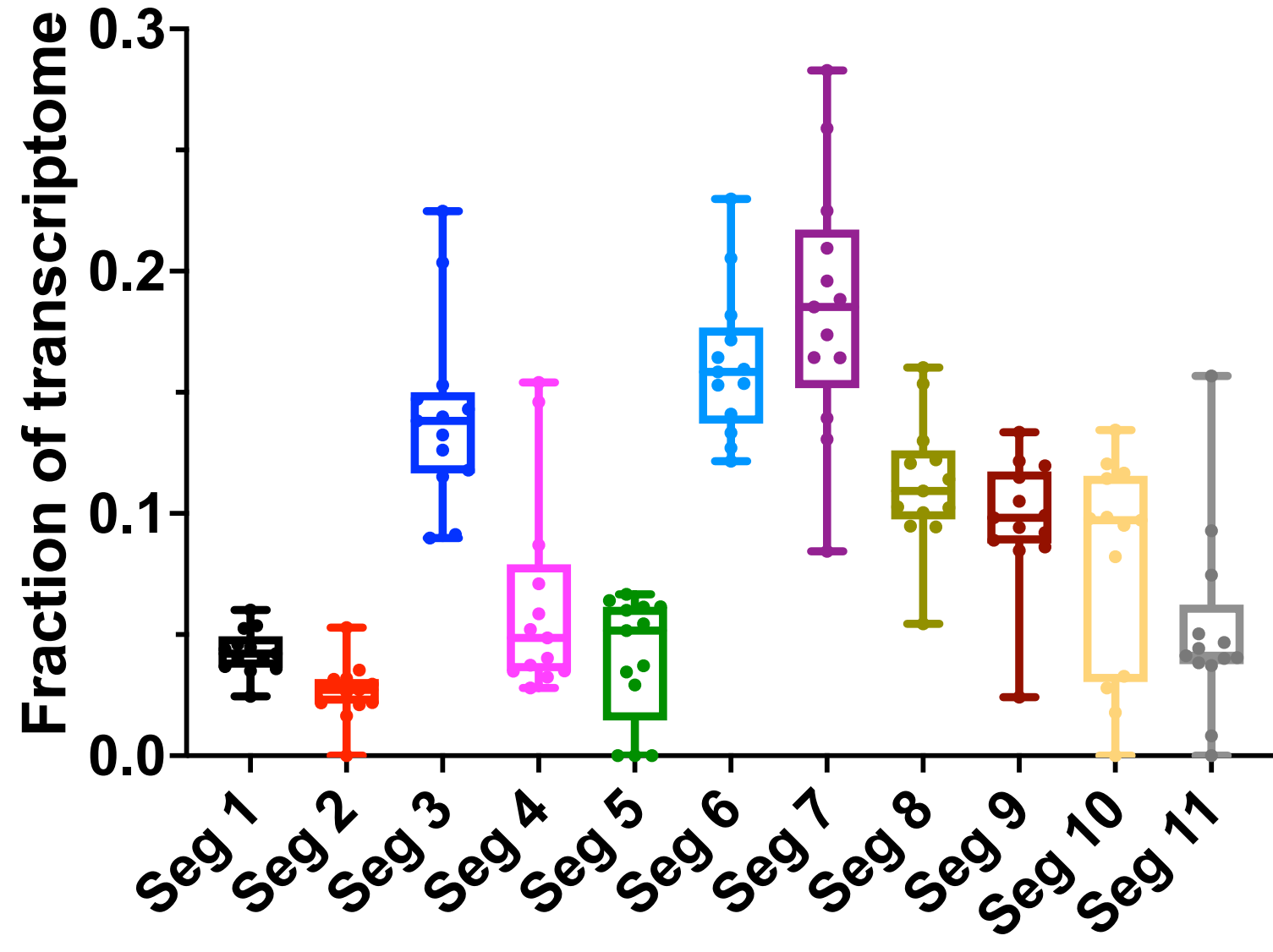

Supplement: Figure 5—source data 2. [file elife-68670-fig5-data2.zip › Fig5 - source_data2_amended_19Jan2023/UDEx-FISH - RNA abundance.pdf]

**Figure 5 source 3**

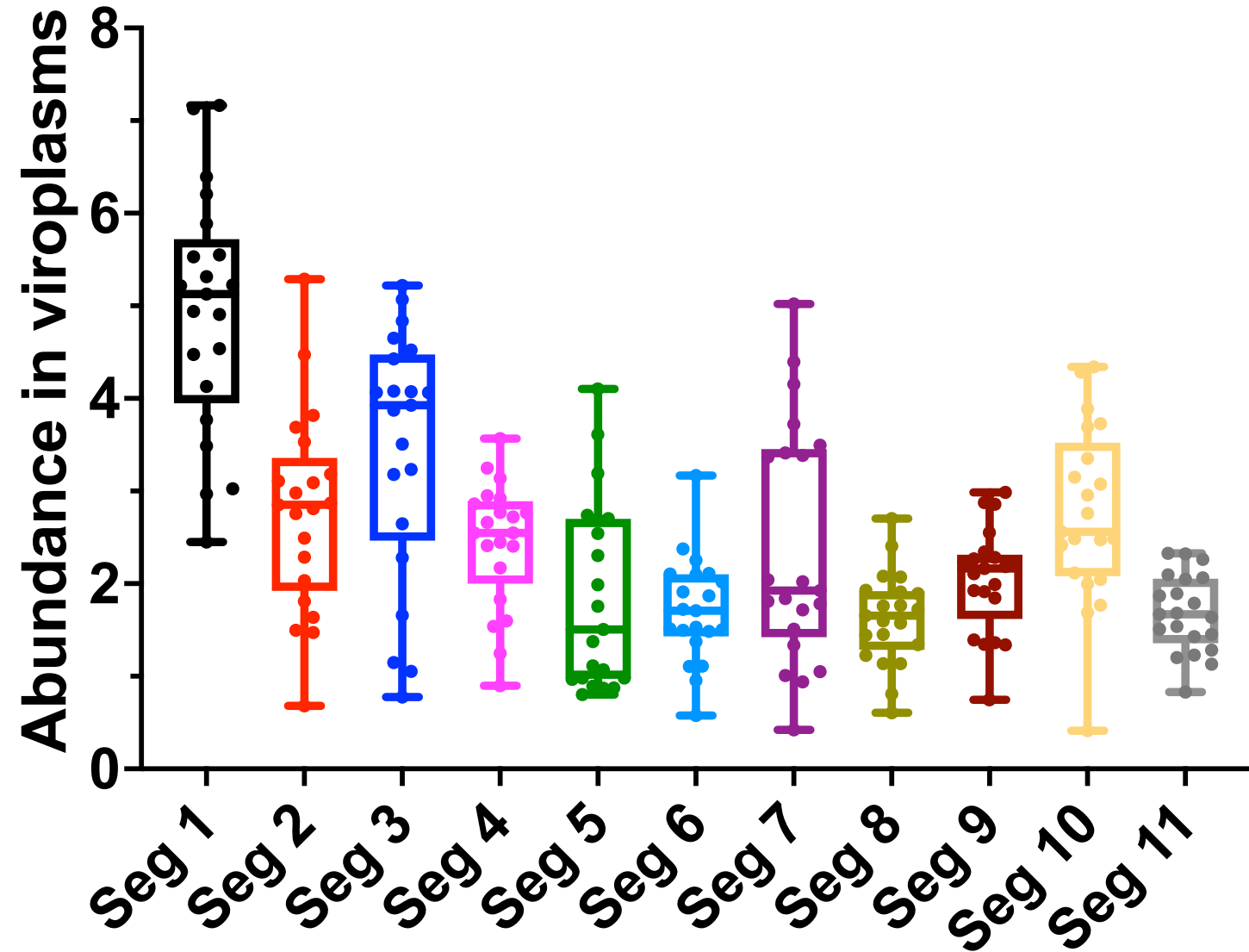

Supplement: Figure 5—source data 3. [file elife-68670-fig5-data3.zip › Fig5 - source_data3_amended_19Jan2023/Fig5 - source 3.pdf]

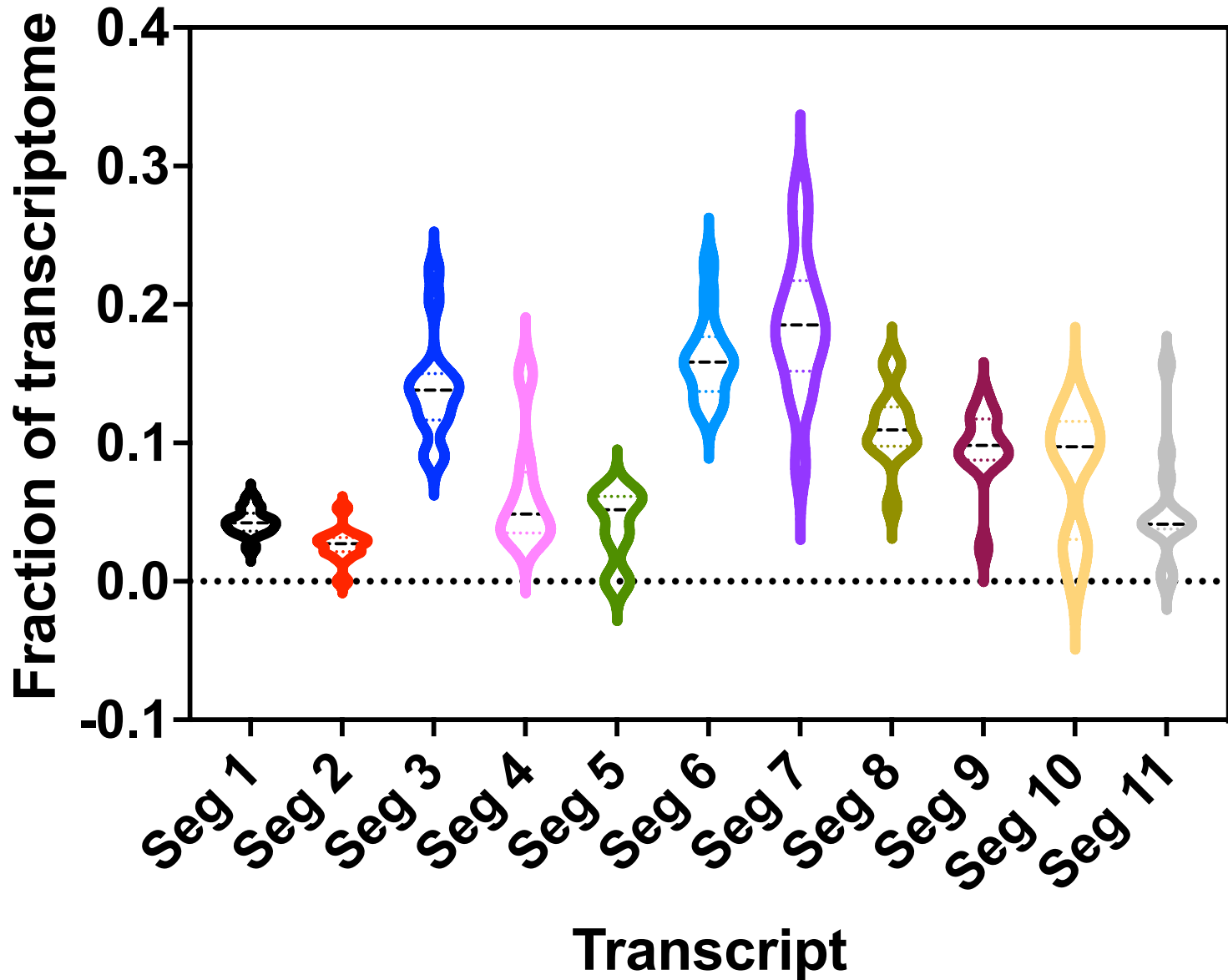

Supplement: Figure 5—source data 4. [file elife-68670-fig5-data4.zip › Fig5 - source_data4_amended_19Jan2023/Fig5 - source_data4 - UDEx-FISH - RNA abundance.pdf]
